# Anteflexion of the Uterus as a Normal Condition Prior to Pregnancy

**Published:** 1853-11

**Authors:** 


*Anteflexion of the Uterus as a normal condition prior to Pregnancy.* By M. Boulard.—A few years since, a very animated discussion took place at the Academie de Médecine, upon the subject of engorgement of the uterus. M. Velpeau was one of those who maintained that anteflexions of the uterus are often mistaken for engorgements, while other speakers declared anteflexion itself to be a very rare occurrence. M. Boulard, engaged in searching for examples of the deviation for Velpeau, was at once struck by the frequency of its occurrence in young subjects, while in older ones he scarcely ever met with a case, without, from the examination of the other organs, ascertaining that the subject of it had never been pregnant. After in this way collecting a great number of uteri from the dead-house, M. Boulard resolved to study the point during the development of the organ, and found that the uterus is almost always anteflexed in the fœtus. He has continued to pursue the investigation from that period; and the present paper is founded upon the examination of 27 adult female subjects who had never borne children, 19 young girls from two to thirteen, and 57 full-timed fœtuses. In 98 of these anteflexion has been found, and to such a point is the body bent upon the neck, that it is not possible to prevent its regaining the same position immediately after the attempt at replacing it has been made. Since these observations have been made, the author has sought every opportunity of verifying them in the living subject; but he has only had ten opportunities of examining the position of the uterus in the virgin, and in all of these the anteflexion existed.

M. Boulard accounts for this disposition having been overlooked by anatomists, by the fact that the bodies of women who have borne one or more children are those which are usually brought for dissection; and the organ is examined only after it has undergone this physiological change, or under the influence of the relaxed state of the tissues after death. When anteflexion has been noted, it has been considered as an abnormal and diseased condition, and regarded as a cause of suffering which was due really to some morbid condition which had become accidentally associated with it.—*Brit. and For. Med. Chir Rev. from Rev. Med. Chir.*, xiii. 341.

